# Adduction spasmodic dysphonia, vocal tremor and muscular tension dysphonia: is it possible to reach a differential diagnosis?

**DOI:** 10.1016/S1808-8694(15)30985-X

**Published:** 2015-10-19

**Authors:** Rui Imamura, Domingos H. Tsuji

**Affiliations:** * PhD, assistant physician at the Otorhinolaryngology Department – University Hospital - Medical School – University of São Paulo; ** Associate Professor – Medical School – University of São Paulo

There may not be a better time to talk about dysphonias then amidst the World Soccer Cup, when offices of otorhinolaryngologists are crowded with aphonic and dysphonic patients because of vocal-abuse laryngitis. Diagnosis in cases like these do not pose major difficulties, a careful interview coupled to a thorough and detailed physical exam are just enough.

In this editorial we shall focus the most challenging topic in laryngology: the differential diagnosis of adduction spasmodic dysphonia, which includes vocal tremor and muscular tension dysphonia. Despite different etiologies, vocal symptoms are very similar in these cases, and the diagnosis can be very subjective, depending a great deal on the personal experience of the examiner.

Adduction spasmodic dysphonia is a vocal disorder characterized by spasms of the laryngeal muscles during speech, causing broken, tense, forced and strangled voice patterns^1^, ^2^. Such disease, classified as central-origin-focal dystonia, of still unknown etiology, remains as one of the most difficult to treat dysphonia type^3^. Its symptoms arise from the intermittent and involuntary contraction of thyroarythenoid muscles during speech, and these result in tense vocal cords, one pushed against the other, and in an increase in glottal resistance^1^, ^4^. It usually starts in patients after their third decades of life, and is more frequent in women. Voice quality usually worsens during stress and improves with sedatives such as alcohol and benzodiazepins^5^. It may cause a negative impact on the life quality of the patient, causing social isolation^6^.

Vocal tremor is characterized by a rhythmic laryngeal movement, causing rhythmic pitch and loudness alterations during speech. These laryngeal movements are often times followed by a tremor of the head and limbs^7^. Tremor may be present only during speech or may happen also during rest. Despite being spotted during cadence speech in some individuals, it is more commonly seen during vocalization of a sustained vowel. Severe cases of vocal tremor may cause speech breaks similar to those of adduction spasmodic dysphonia^8^.

Tension dysphonia is associated to excessive muscular tension, both in the intrinsic and extrinsic laryngeal muscles, with an over adduction of the true or false vocal cords, and even sphincteric laryngeal constriction. This hyperfunctional pattern usually persists in various phonatory situations^7^, ^8^.

The differentiation among these three entities is not always easy for otorhinolaryngologists, since their main diagnostic tool, the laryngoscopic exam, does not show characteristic structural changes in any of the cases. To make matters even worse, there may be an occasional association between these conditions. For instance, patients with spasmodic dysphonia may present vocal tremor or muscle tension dysphonia associated^8^.

Since there are no clear morphological findings at the exam, many patients end up not being properly diagnosed and are eventually considered bearers of conversion or psychiatric disorders by family and physicians. Treated as such, often times they spend years without improvement. The psychological repercussion accruing from this situation may be catastrophic for the patient's social relations.

Even for experienced laryngologists, used to dealing with disorders like this, diagnosis can be challenging. Incorrect diagnosis may lead to a less successful treatment. For instance, the use of Botulin toxin, treatment modality recommended for spasmodic dysphonia, for a patient with muscle tension dysphonia, may cause severe glottal incompetence, with severe hoarseness and aspiration of saliva and food^8^.

Because of the challenging diagnosis of this condition, many investigators have studied methods that assess these patients in a more objective fashion. It is within this context that this month's issue brings the original paper “Adduction laryngeal dystonia: proposal and nasofibrolaryngoscopy protocol evaluation”. We still need other studies that may provide solid basis that allow us to carry out the differential diagnosis of spasmodic dysphonias in a more efficient way, and all studies in this sense are, of course, very much welcome.
